# Iron Deficiency Anaemia: Its Prevalence Among Women of Reproductive Age in Shanghai and Tokyo and Links to Body Mass Index

**DOI:** 10.7759/cureus.9436

**Published:** 2020-07-28

**Authors:** Kana Yamamoto, Na Wang, Morihito Takita, Yuto Maeda, Tetsuya Tanimoto, Andy Crump, Yonggen Jiang, Genming Zhao

**Affiliations:** 1 Internal Medicine, Navitas Clinic, Tokyo, JPN; 2 Epidemiology, School of Public Health, Key Laboratory of Public Health Safety of Ministry of Education, Fudan University, Shanghai, CHN; 3 Obstetrics and Gynecology, Center for Maternal-Fetal, Neonatal and Reproductive Medicine, National Center for Child Health and Development, Tokyo, JPN; 4 Epidemiology and Public Health, Graduate School of Infection Control Sciences, Kitasato University, Tokyo, JPN; 5 Epidemiology, Songjiang Center for Disease Prevention and Control, Shanghai, CHN

**Keywords:** asia, anaemia, body mass index, iron deficiency, women, thinness

## Abstract

In this study, we examined the prevalence of iron deficiency anaemia (IDA) among young women between the ages of 20-44 living in Shanghai, China, and Tokyo, Japan with a particular emphasis on investigating its possible links with body mass index (BMI). Shanghai data were obtained from annual health check-up records conducted between May and September 2016 (n=2,006). Tokyo data were from health examinations of employees working in dispensing pharmacies between July and February 2017 (n=877). Anaemia prevalence was found to be 14.8% and 11.4% in Shanghai and Tokyo, respectively. The proportion of women with a BMI of <18.5 was 9.9% in Shanghai and 25.7% in Tokyo. Anaemia prevalence tends to be high in women with a low BMI. Women with a BMI of <18.5 had the highest prevalence of anaemia (18.2%) in Shanghai. Yet, the prevalence of anaemia was low (9.3%) among Tokyo women with a BMI of <18.5, significantly lower than those with a BMI between 18.5-25.0 (12.5%). IDA is a significant problem among women in both Shanghai and Tokyo, where the ‘desire for thinness” is commonplace among young women. The Tokyo participants with a low BMI, however, appear to have managed to avoid developing IDA.

## Introduction

The World Health Organization (WHO) estimates that some two billion people are anaemic worldwide; anaemia is defined as having blood haemoglobin (Hb) concentrations below normal thresholds [1]. It is a condition in which the number of red blood cells (RBC) and their oxygen-carrying capacity are insufficient to meet the body’s physiological needs. These needs vary with a person’s age, gender, residential elevation above sea level (altitude), smoking behaviour, different stages of pregnancy and socioeconomic status. The main causes of anaemia are dietary iron deficiency; blood loss due to mensuration and cancer; infectious diseases (such as malaria, hookworm and schistosome infections); deficiencies of other micronutrients, including folate, vitamin B12 and vitamin A; inherited or acquired disorders that affect RBCs, such as thalassaemia; and, increasingly, human immunodeficiency virus (HIV) infection/acquired immunodeficiency syndrome (AIDS). Iron deficiency is the most common cause of anaemia globally. Iron deficiency anaemia (IDA) and iron deficiency are major global health concerns, affecting more than two billion people worldwide in both developed and developing countries [2]. Iron deficiency causes weakness, fatigue, difficulty in concentrating, and poor educational performance or work productivity due to non-specific symptoms ascribed to the diminished levels of oxygen delivered to body tissues. IDA also affects maternal and child mortality, with infants aged zero to five years, women of childbearing age, and pregnant women particularly at risk. Several chronic diseases are also linked with IDA, including chronic kidney disease, chronic heart failure, cancer and inflammatory bowel disease. Moreover, IDA in pregnant women has been proved to be particularly dangerous, leading to increases in maternal mortality, perinatal mortality and premature delivery. Since 2010, the rates of anaemia among women of reproductive age have been steadily increasing in both Japan and China [3], and this report is a preliminary study to investigate this phenomenon.

Several factors, including menstrual bleeding and insufficient intake of iron, have been reported to cause IDA; the underlying mechanisms of IDA is not yet fully understood. Insufficient dietary intake is the main cause in most low-income countries, whereas excessive consumption of food and some specific eating habits, such as a vegetarian diet or avoidance of red meat, frequently lead to reduced iron intake, subsequently causing IDA in industrialised countries [2]. In addition, the lifestyles of young women have lately been undergoing significant changes in many countries, particularly relating to the social advancement of women, including spending more time in higher education, marrying later, with concomitant increases in later-age births and a declining birth rate, as well as the societal pressure to maintain a modern, fashionable and lissom frame. A considerable number of women restrict their nutritional intake in pursuit of a lean body image promoted by social influences [4]. This trend is particularly prominent in East Asia. In Japan, the declining weight of women has been regarded as a problem since World War II [5]. The number of women with low body weight increased from 4.4 million in 1975 to 5.7 million in 2014 [6]. These women tend to take care of their health and outward appearance and use large amounts of supplements, including iron [7]. Similarly, many women in China strive to have a slim figure [8], and their body mass index (BMI) has also generally decreased over the last 20 years. These women subsequently may have a higher risk of IDA, although there has been little evidence published to support this assumption.

To find out whether there is indeed a link between BMI decline in young women and IDA, we examined recent records of health examinations of women living in Shanghai and Tokyo to elucidate the prevailing status of IDA in these two megacities.

## Materials and methods

Definitions

In the absence of an international agreement on how to assess the iron status of populations, the prevalence of iron deficiency has often been derived from the prevalence of anaemia by using measurements of blood Hb concentration. However, not all anaemic people are iron deficient and iron deficiency may occur without anaemia. Hb concentration alone cannot be used to diagnose iron deficiency; however, due to the limitations of this study, no data was gathered on other diagnostic factors such as zinc protoporphyrin, transferrin receptor and serum ferritin levels. Nevertheless, Hb concentration measurement can provide an important indicator of the severity of iron deficiency [2]. For the purposes of this study, anaemia was defined as a condition in which the concentration of Hb in blood was lower than 12.0 g/dL, in accordance with the WHO criteria [9]. Severe, moderate and microcytic anaemia were defined as having Hb levels of <10 g/dL; ≤10 g/dL and <12 g/dL; plus mean corpuscular volume (MCV) of <80 fL, respectively. MCV indicates whether RBCs are smaller than usual (microcytic), which is a common sign of IDA. The RBC count was measured at the International Standards Organization (ISO)-accredited laboratories in both countries.

Data collection

Data for Shanghai were obtained from the records of annual health check-up programmes conducted between May and September 2016 for residents in Xinqiao, Sheshan and Maogang towns in the Songjiang District of Shanghai (n=2,006). The three towns are located in the southwestern part of Shanghai, and their population was approximately 230,000 in total according to the Sixth National Population Census of the People’s Republic of China.

Data for Tokyo were obtained from health examinations of employees working in several dispensing pharmacies in Tokyo between July and February 2017 (n=877). We included data from women between the ages of 20-44 years old. Data obtained from women receiving treatments for haematological diseases were excluded. The data was provided by administrative offices involved in the health examination programmes. The study was approved by the Institutional Review Board of the Medical Governance Research Institute in Tokyo, Japan (approval number: MG2018-01).

Statistical analysis

A correlation was evaluated with Spearman’s correlation coefficients. A p-value of <0.05 was considered significant. The statistical analysis was performed with JMP 13.2.1 (SAS Institute Inc., Cary, NC).

## Results

Prevalence of anaemia in Shanghai and Tokyo

Participants’ characteristics are shown in Table 1. A diagnosis of anaemia was established in 297 (14.8%) and 100 (11.4%) participants in Shanghai and Tokyo, respectively. The prevalence of severe anaemia was less than 3% in both cohorts: 53 (2.6%) and 12 (1.4%) in Shanghai and Tokyo, respectively. Microcytic anaemia was found in 115 (5.7%) and 30 (3.4%) women in Shanghai and Tokyo, respectively.

**Table 1:** Participants' characteristics and prevalence of anaemia IQR: interquartile range

	Shanghai cohort (n=2,006)		Tokyo cohort (n=877)	
Participants' characteristics and blood cell count	Median (range)	IQR	Median (range)	IQR
Age (years)	36 (20–44)	13	30 (21–44)	8
Body mass index, kg/m^2^	21.7 (14.7–41.5)	4.06	20.5 (13.3–42.8)	3.2
Haemoglobin, g/dL	13.1 (6.4–17.9)	1.4	13.0 (8.7–16.0)	1.3
Red blood cell count, ×10^9^/L	4.5 (3.2–6.4)	0.46	4.4 (3.3–5.5)	0.45
Hematocrit, %	40.6 (26.6–55.8)	3.7	40.0 (29.2–48.3)	4.0
Mean corpuscular volume, fl	91.6 (59.0–107.2)	6.3	91 (61.0–106.0)	6.0
Mean corpuscular haemoglobin concentration, g/dL	31.8 (18.6–35.0)	1.7	32.8 (28.4–36.3)	1.6
White blood cell count, ×10^9^/L	5.8 (2.1–16.4)	2.0	5.5 (2.8–11.7)	2.0
Platelet count, ×10^9^/L	223 (55.0–592.0)	77	251 (99.0–494.0)	70
Prevalence of anaemia	n (%)		n (%)	
Anaemia	297 (14.8)		100 (11.4)	
Moderate anaemia	244 (12.2)		88 (10.0)	
Severe anaemia	53 (2.6)		12 (1.4)	
Microcytic anaemia	115 (5.7)		30 (3.4)	
Mentzer index of <13	9 (0.5)		3 (0.3)	

Prevalence of anaemia classified by age group

**Figure 1:**
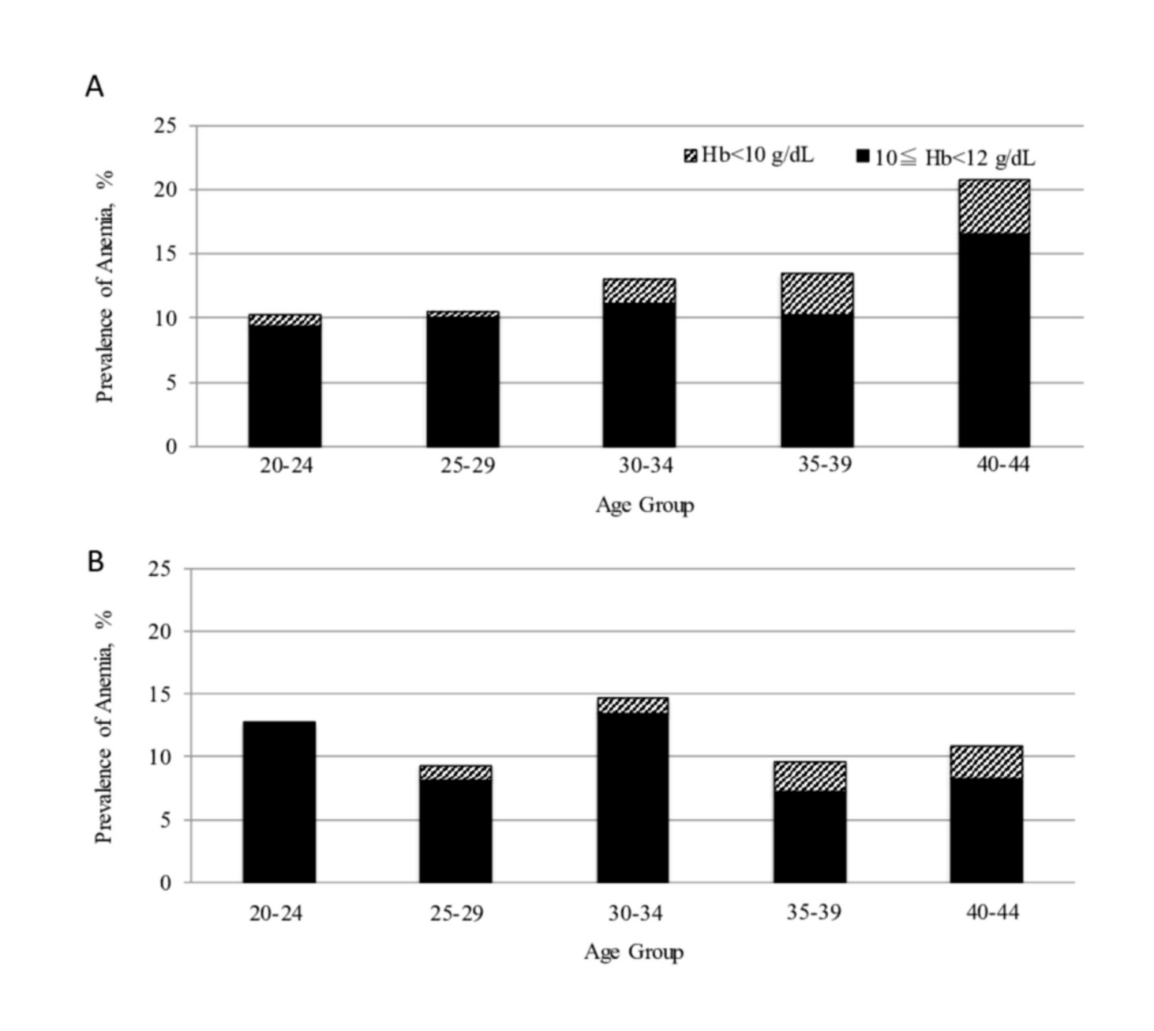
Prevalence of anaemia by age group Prevalence of anaemia and severe anaemia are shown by age group in Shanghai (A) and Tokyo (B) cohorts

The prevalence of anaemia by age groups is shown in Figure 1. The highest prevalence of anaemia was seen in participants of ≥40 years old in the Shanghai cohort, whereas it was found in those of 30-34 years old in Tokyo. A significant association was found between the participants’ age and prevalence of anaemia in Shanghai [Spearman rank correlation coefficient ρ=1.0 (p: <0.0001)], but not in Tokyo. Severe anaemia was most frequently seen among participants of 40-44 years old in both cohorts (4.3 and 2.7% in Shanghai and Tokyo, respectively) (Table 2).

**Table 2:** Prevalence of anaemia by age group

	Shanghai cohort	Tokyo cohort
Prevalence of anaemia in total (%)		
20–24 years	10.3	12.8
25–29	10.4	9.3
30–34	13	14.7
35–39	13.5	9.6
40–44	20.8	11
Prevalence of moderate anaemia (%)		
20–24 years	9.4	12.8
25–29	10.1	8.1
30–34	11.2	13.4
35–39	10.3	7.2
40–44	16.6	8.2
Prevalence of severe anaemia (%)		
20–24 years	0.9	0
25–29	0.3	1.2
30–34	1.8	1.3
35–39	3.2	2.4
40–44	4.3	2.7

Prevalence of anaemia by BMI group

**Figure 2:**
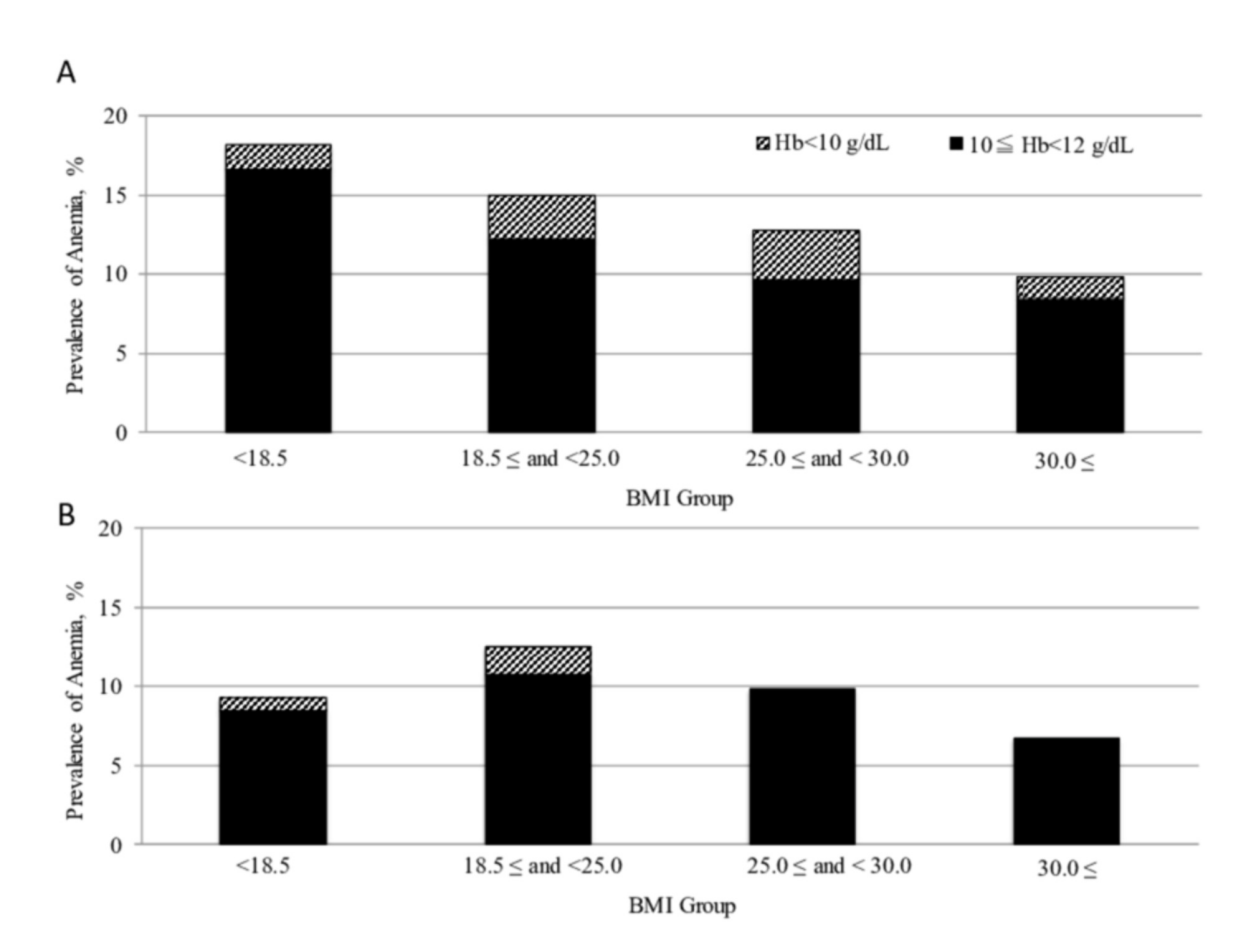
Prevalence of anaemia by BMI group Prevalence of anaemia and severe anaemia are shown by BMI groups in Shanghai (A) and Tokyo (B) cohorts BMI: body mass index

The proportion of participants with a BMI of <18.5 was 9.9% in Shanghai and 25.7% in Tokyo, as shown in Figure 2. In Shanghai, the highest prevalence of anaemia was seen in participants with a BMI of <18.5, with the prevalence of anaemia generally decreasing as BMI increased. Conversely, in Tokyo, the prevalence of anaemia was unexpectedly lower in those with a BMI of <18.5 compared to participants with a BMI between ≥18.5 and <25.0 (Table 3).

**Table 3:** Prevalence of anaemia by BMI group BMI: body mass index

	Shanghai cohort	Tokyo cohort
Prevalence of anaemia (%)		
BMI of <18.5 kg/m^2^	18.2	9.3
≥18.5 and <25.0	15	12.5
≥25.0 and <30.0	12.8	9.8
≥30.0	9.9	6.7
Prevalence of moderate anaemia (%)		
BMI of <18.5 kg/m^2^	16.7	8.4
≥18.5 and <25.0	12.2	10.8
≥25.0 and <30.0	9.7	9.8
≥30.0	8.5	6.7
Prevalence of severe anaemia (%)		
BMI of <18.5 kg/m^2^	1.5	0.8
≥18.5 and <25.0	2.8	1.7
≥25.0 and <30.0	3.1	0
≥30.0	1.4	0

Median haemoglobin levels by age group and BMI group

**Figure 3:**
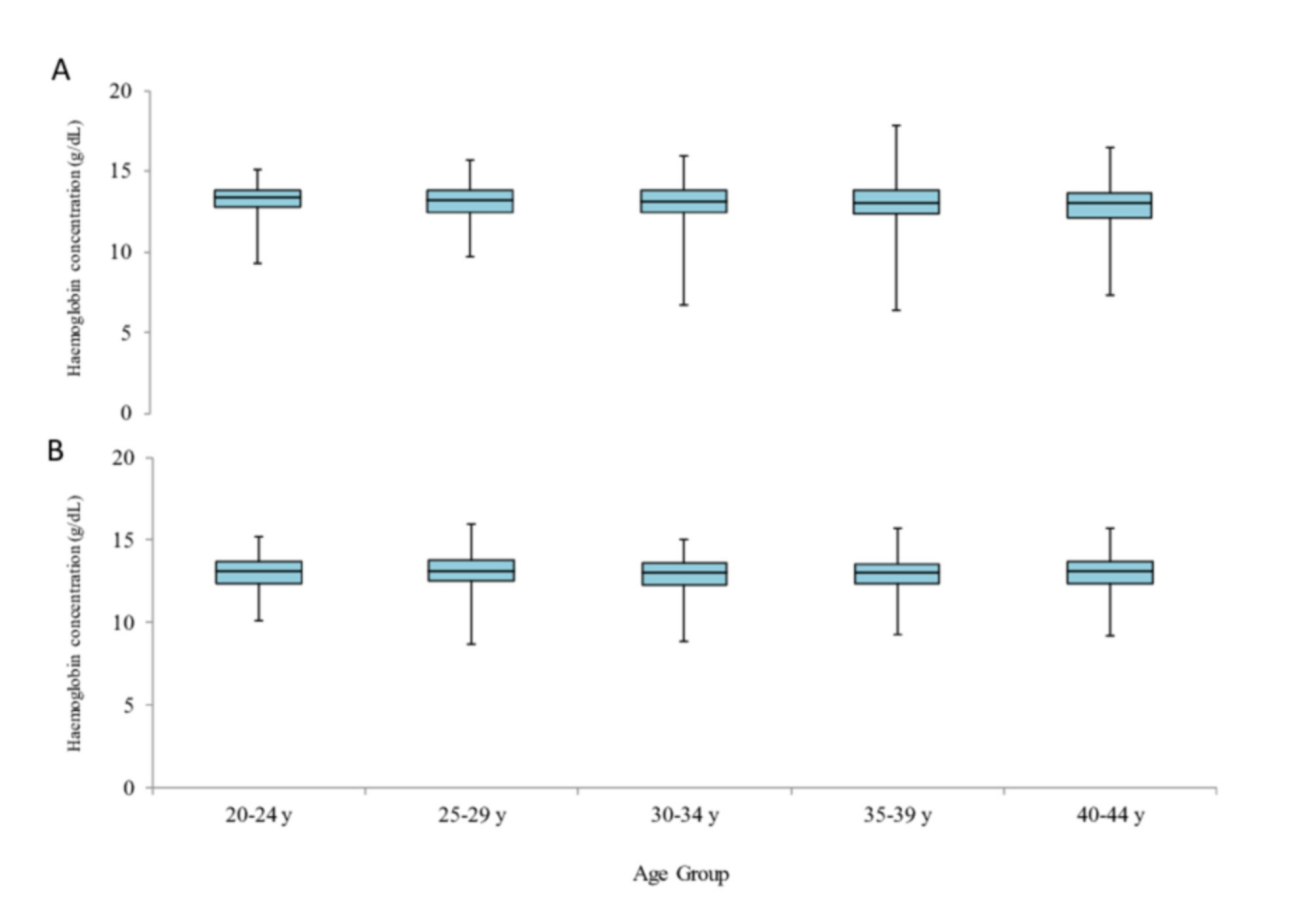
Haemoglobin levels by age group Levels of haemoglobin are shown by age group in Shanghai (A) and Tokyo (B) cohorts. Box plots are shown as median values, interquartile ranges and ranges excluding outliers that are above or below 1.5 times the interquartile range

**Figure 4:**
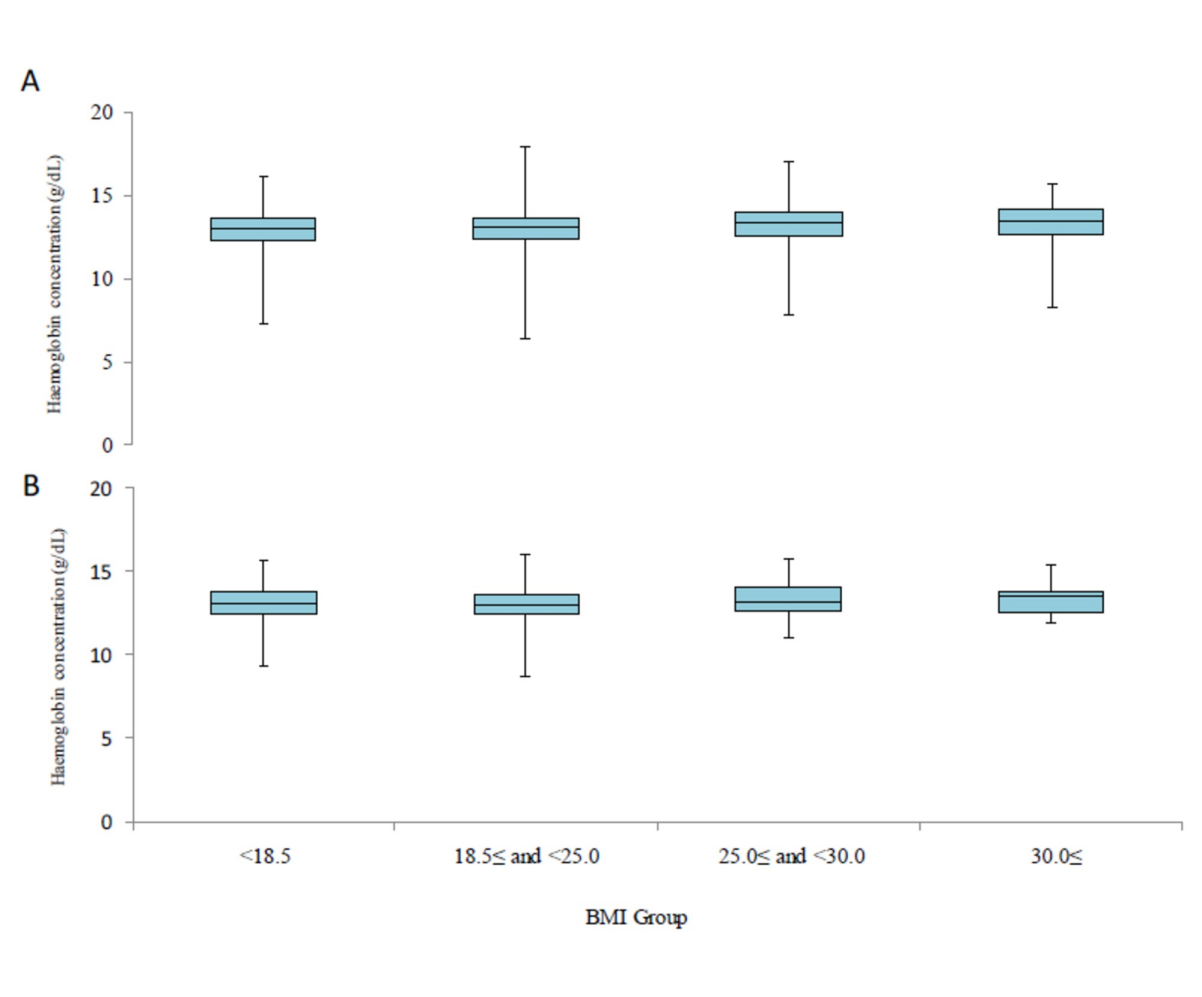
Haemoglobin levels by BMI group Levels of haemoglobin are shown by BMI group in Shanghai (A) and Tokyo (B) cohorts. Box plots are shown as median values, interquartile ranges and ranges excluding outliers that are above or below 1.5 times the interquartile range BMI: body mass index

The median Hb levels according to age and BMI groups are shown in Figures 3, 4, respectively. The median value of Hb was similar between both these subgroups.

Platelet and white blood cell (WBC) counts

WBC and platelet count for participants in Shanghai and Tokyo are shown in Table 1.

## Discussion

This study showed that anaemia may well be an increasingly important health issue among young women living in Tokyo and Shanghai who follow a lifestyle where they try to maintain a slim, sylphlike body image. The prevalence of anaemia was 14.8% in Shanghai and 11.4% in Tokyo, which are much higher compared with those in the United States (5.6%) [10] and Europe (2-5%) [11]. These figures indicate that the prevalence of anaemia among those individuals studied indicates a “Normal” (≤4.9%) to “Mild” (5.0-19.9%) level of public health significance, according to WHO criteria [9].

There are two major causes of anaemia in young females: insufficient red cell production due to poor intake of dietary iron and excessive blood loss through menstruation. Notably, dietary intake of iron is suboptimal in Japan and China. The Japanese ingest an average of 6.5 mg/day of iron [12], which is lower compared to the United States (12.6-13.5 mg/day) [13] and the United Kingdom (9.8 mg/day) [14]. Iron intake in China exceeds these totals (17.7 mg/day) [15]. However, iron ingested in China is mainly non-haem (16.2 mg/day), which is poorly absorbed from the digestive tract. Despite the rapid advance in the Chinese economy and standard of living, the main ingredients in Chinese food are cereals, potatoes and vegetables, and consumption of meat, which is rich in haem iron, is low compared with that of developed countries [15].

Iron deficiency is a common finding in women with heavy menstrual bleeding. Long-term oral contraceptive use is effective for reducing blood loss through menstruation. However, the rates of contraceptive pill usage are 1.2% in China and 0.9% in Japan, far lower than that of the United Kingdom (28.0%) and United States (13.5%) where the prevalence of anaemia is lower [16]. Consequently, the low usage of contraceptive pills may have a significant effect on iron deficiency in women in Shanghai and Tokyo.

The prevalence of anaemia was higher in Shanghai than in Tokyo, and there are several reasons to help explain these findings. Firstly, Shanghai attracts approximately six million migrants annually, and the influx of citizens from elsewhere in China accounts for about a quarter of the total population of the city [17]. The prevalence of anaemia associated with an inadequate intake of iron is high in rural areas in China compared with Shanghai [18]. Furthermore, thalassaemia, which is classified as small spherical hypochromic anaemia, is common in China, with a prevalence of 10.6% [19], over 100-fold of the level in Japan (0.1%) [20]. Taken together with the dietary difference, it is reasonable to assume that these findings contribute to elevating the prevalence of anaemia in Shanghai.

The results identified two key differences between participants in Shanghai and those in Tokyo. The anaemia profile in China showed an increasing prevalence with age, especially with respect to severe anaemia (Figure 1A), whereas that in Tokyo did not (Figure 1B). In addition, the profile of anaemia in China displays a decreasing trend with increasing BMI, while that is not the case in Tokyo (Figures 2A, 2B). Of note, the national averages of BMI for females of 18 years of age and older were reported as 23.7 and 22.1 kg/m^2^ in China and Japan, respectively, in 2016 [21]. There was no significant difference between the cohorts in this study and the national surveys; however, the medians of BMI in this study are slightly lower than the national averages. The socioeconomic characteristics of urban megacities may have influenced these results.

The association between anaemia and BMI, which is effectively a measure of the nutritional and health status of adults [22], remains a matter of conjecture. Previous studies have reported the occurrence of anaemia in both under-nourished and over-nourished individuals, loosely representing the poor and wealthy socioeconomic classes, respectively [2], whereas other studies have associated anaemia with a low BMI [23]. Our study indicated that anaemia prevalence tended to be high in women with a low BMI in Shanghai but not in Tokyo. The Shanghai observation supports findings in other studies undertaken in China, Peru, Egypt and the United States, which indicated that anaemia displayed a decreasing trend with increasing BMI [24,25]. But this contrasts with the findings from studies in Nigeria and Mexico, which found no definite relationship between IDA and BMI [26,27]. Of note, obesity has been reported as a potent risk factor for iron deficiency as a consequence of inflammatory status mediated by hepcidin [28]. The results showing the lowest prevalence of anaemia in participants with a BMI of ≥30 in both countries does not align with the concept of hepcidin-regulated anaemia. In the general population, dietary insufficiency of iron for erythropoiesis may outweigh obesity-induced inflammatory status as a cause of anaemia.

Our findings are consistent with the fact that IDA frequently results from an insufficient intake of dietary iron, which is usually linked with total nutritional intake. It should be noted that the average BMI of Japanese women has decreased since the end of World War II [5]. The ratio of women with a BMI of <18.5 was 25.7% in Tokyo in the present study, much higher than that in Shanghai (9.9%), and comparable with developing countries such as India, where 25% of women are underweight [6].

Many young women’s desire to stay slim, and the ‘desire for thinness’ is a significant problem in Asian countries. However, few studies have reported the association between anaemia and slenderness. Surprisingly, the prevalence of anaemia in Tokyo women with a BMI <18.5 was unexpectedly lower than in those with a BMI of ≥18.5 and <25.0. Japanese women might adapt their food intake or take additional supplements to keep healthy by themselves. This may well be the case among the women in this study, who worked at pharmacies where dietary supplements are sold. They might take foods containing a large amount of haem iron, such as fish and pork, or iron supplements while simultaneously limiting their calorie intake. Having a clinical eating disorder, such as anorexia or bulimia, may cause a woman’s menstruation to stop, which often happens as a result of a nutritional deficiency or very low body weight. Nakai et al. have reported a prevalence of 12.7% of eating disorder among Japanese female students aged between 16 and 23 years, which is higher than the findings of Chinese and global surveys (2.2% for females aged 12 and 22 in China and 8.4% for females in global metanalysis) [29,30]. In Japan, the use of contraceptive pills is relatively low and does not significantly impact blood loss during menstruation. Further studies are warranted to investigate these issues and how they may interact with each other.

The study faced some severe limitations. Firstly, the methods of recruitment of the participants in Shanghai and Tokyo were different; data on health examination for residents was used in Shanghai but not in Tokyo, where data on physical examination of pharmacy workers was collected. It is far more likely that pharmacy workers may possess medical knowledge on the prevention and treatment of IDA; moreover, they were earning an income and so there may be several disruptive differentials complicating comparison between the two groups. Secondly, since the participants in this study voluntarily underwent medical examinations, there is a possibility that they might have strong interests in following a healthy lifestyle and being watchful for the symptoms of anaemia. Moreover, Japanese women have the benefit of access to an excellent health service and universal health insurance. Furthermore, individuals not receiving a full medical examination might have a lower incidence of IDA than the participants in this study. In addition, the data of characteristics obtained in this study were limited to age and BMI, and other critical factors, such as pregnancy, iron supplement use, frequency of blood donation and medical histories, could not be obtained. Collecting data on more of these elements, including food habits and socioeconomic backgrounds, will be essential to properly and accurately measure factors influencing IDA.

## Conclusions

IDA may soon become a more serious public health problem, especially in women of reproductive age, in both Japan and China, especially if its prevalence continues to rise. Considering the association observed between a low BMI and the prevalence of anaemia, insufficient dietary intake of iron may well be a primary cause of the condition in the two cities studied. Significantly, the study indicated that Japanese young women might have succeeded in reducing their weight in a healthy fashion with no apparent appearance of IDA. Further large-scale studies are warranted to clarify and confirm this interesting finding and to help reduce the negative public health impacts of IDA in China and Japan.
